# Development of a PROTAC-Based Targeting Strategy Provides a Mechanistically Unique Mode of Anti-Cytomegalovirus Activity

**DOI:** 10.3390/ijms222312858

**Published:** 2021-11-27

**Authors:** Friedrich Hahn, Stuart T. Hamilton, Christina Wangen, Markus Wild, Jintawee Kicuntod, Nadine Brückner, Jasmine E. L. Follett, Lars Herrmann, Ahmed Kheimar, Benedikt B. Kaufer, William D. Rawlinson, Svetlana B. Tsogoeva, Manfred Marschall

**Affiliations:** 1 Institute for Clinical and Molecular Virology, Friedrich-Alexander University of Erlangen-Nürnberg (FAU), 91054 Erlangen, Germany; christina.wangen@uk-erlangen.de (C.W.); markus.wild@uk-erlangen.de (M.W.); jintawee.kicuntod@extern.uk-erlangen.de (J.K.); nadine.brueckner@fau.de (N.B.); 2 Serology and Virology Division, NSW Health Pathology Microbiology, Prince of Wales Hospital, Schools of Women’s and Children’s Health, Medicine and Biotechnology and Biomolecular Sciences, University of New South Wales, Sydney, NSW 2031, Australia; stuart.hamilton@health.nsw.gov.au (S.T.H.); j.follett@student.unsw.edu.au (J.E.L.F.); w.rawlinson@unsw.edu.au (W.D.R.); 3 Institute of Organic Chemistry I, FAU, 91058 Erlangen, Germany; lars.herrmann@fau.de (L.H.); svetlana.tsogoeva@fau.de (S.B.T.); 4 Institute of Virology, Freie Universität Berlin, 14163 Berlin, Germany; ahmed.kheimar@fu-berlin.de (A.K.); benedikt.kaufer@fu-berlin.de (B.B.K.)

**Keywords:** human cytomegalovirus, human/animal pathogenic viruses, antiviral drugs, direct-acting antivirals, host-directed antivirals, PROteolysis TArgeting Chimeras, PROTAC-based targeting strategy, new drug qualities

## Abstract

Human cytomegalovirus (HCMV) is a major pathogenic herpesvirus that is prevalent worldwide and it is associated with a variety of clinical symptoms. Current antiviral therapy options do not fully satisfy the medical needs; thus, improved drug classes and drug-targeting strategies are required. In particular, host-directed antivirals, including pharmaceutical kinase inhibitors, might help improve the drug qualities. Here, we focused on utilizing PROteolysis TArgeting Chimeras (PROTACs), i.e., hetero-bifunctional molecules containing two elements, namely a target-binding molecule and a proteolysis-inducing element. Specifically, a PROTAC that was based on a cyclin-dependent kinase (CDK) inhibitor, i.e., CDK9-directed PROTAC THAL-SNS032, was analyzed and proved to possess strong anti-HCMV AD169-GFP activity, with values of EC_50_ of 0.030 µM and CC_50_ of 0.175 µM (SI of 5.8). Comparing the effect of THAL-SNS032 with its non-PROTAC counterpart SNS032, data indicated a 3.7-fold stronger anti-HCMV efficacy. This antiviral activity, as illustrated for further clinically relevant strains of human and murine CMVs, coincided with the mid-nanomolar concentration range necessary for a drug-induced degradation of the primary (CDK9) and secondary targets (CDK1, CDK2, CDK7). In addition, further antiviral activities were demonstrated, such as the inhibition of SARS-CoV-2 replication, whereas other investigated human viruses (i.e., varicella zoster virus, adenovirus type 2, and Zika virus) were found insensitive. Combined, the antiviral quality of this approach is seen in its (i) mechanistic uniqueness; (ii) future options of combinatorial drug treatment; (iii) potential broad-spectrum activity; and (iv) applicability in clinically relevant antiviral models. These novel data are discussed in light of the current achievements of anti-HCMV drug development.

## 1. Introduction

Human cytomegalovirus (HCMV) is the prototype species of *Betaherpesvirinae* and a major opportunistic human pathogen that is prevalent worldwide. Seroprevalence ranges between 40% and 95% in the adult human population, and is dependent on sociogeographical factors. HCMV infection is typically asymptomatic or involves mild symptoms, i.e., of mononucleosis-like diseases in immunocompetent individuals, but could lead to high morbidity and mortality in immunocompromised individuals [1]. Symptomatic infections are seen in patients with conditions treated with immunosuppressive therapy, such as antitumoral chemotherapy, stem cell/organ transplantation, or coinfection with human immunodeficiency virus type 1 (HIV-1). Most importantly, congenital HCMV infection (cCMV) of the unborn is the main infection-based risk during pregnancy [2,3,4]. Thus, cCMV can cause a wide range of symptoms, from mild to severe, or even life-threatening in up to 25% of infected embryos or infants, mainly manifesting as acute or late-onset embryonal developmental defects, such as sensorineural hearing loss [5]. At present, a distinct repertoire of approved anti-HCMV drugs is available for the prevention and control of infection, mostly comprising nucleoside/nucleotide or pyrophosphate analogs that intervene with the activity of viral genome replication. These are ganciclovir (GCV), its oral prodrug valganciclovir (VGCV), foscarnet (FOS), cidofovir (CDV) and, to a lesser extent of efficacy, acyclovir, (ACV) [6,7]. In 2017, letermovir (LMV, Prevymis^®^) has been additionally approved for prophylactic use against HCMV infection in recipients of hematopoietic stem cell transplantation [8,9]. LMV represents the first approved, mechanistically novel terminase inhibitor, which targets the viral terminase complex to interfere with the processing and packaging of viral genomic DNA into mature capsids [10]. However, LMV and all currently approved anti-HCMV drugs, face limitations, such as viral drug resistance and, in many instances, severe side effects. These side effects can include nephrotoxicity, myelotoxicity, and anemia, which limit therapeutic compatibility, particularly in long-term treatments [11,12]. To address the issues of HCMV prevention and treatment, optimized drug application schemes and novel targeting strategies are essential. For this reason, researchers are looking into the development of mechanistically novel antiviral drug candidates and unexploited targeting strategies [13].

In particular, the kinase inhibitor maribavir (MBV) constitutes an anti-HCMV drug candidate that is in the advanced stages of development [14], recently being investigated in four phase III clinical trials (NCT02931539, NCT02927067, NCT00497796, NCT00411645). MBV targets the HCMV-encoded ortholog of cyclin-dependent kinases (CDKs), pUL97, which play important roles in viral replication, particularly the HCMV nuclear capsid [13,15,16,17,18,19,20,21]. This principle of introducing pharmaceutical kinase inhibitors (PKIs) as antivirals, so far well-known and positively experienced in antitumoral therapies, would be new in the entire field of antiviral therapy and is regarded by many investigators as a highly promising development. Nevertheless, it is very likely that (the soon expected) clinical approval of MBV will not resolve the problems of antiviral drug resistance. The resistance barrier of direct-acting antivirals (DAAs) is generally found at a relatively low level, i.e., due to the ease of drug-induced viral mutagenesis. This unfortunate weak point might stand in sharp contrast to the profoundly different targeting situation that host-directed antivirals (HDAs) would probably entail. Promising candidates for this host-directed approach, i.e., PKIs that selectively target human CDKs, already exist, or will be newly synthesized. In our previous studies, inhibitors of CDKs 7, 9, 1, 2, as well as pan-CDK inhibitory compounds, showed very pronounced anti-cytomegaloviral activities, both in vitro and in vivo [22,23,24,25,26,27,28,29]. Notably, these CDK-specific HDAs are proven to have broad-spectrum antiviral activity potential [26]; their usefulness for drug combination approaches has been demonstrated by others and our group [30,31] (for reviews, see Britt and Prichard, 2018 [11] and Steingruber and Marschall, 2020 [13], and references therein). 

To increase the CDK-inhibitory properties and antiviral efficacies of these drugs, we favored the chemical coupling of moieties, termed PROteolysis TArgeting Chimeras (PROTACs), as a modification with potentially promising and drug-optimizing quality. The concept suggests that PROTAC-coupled HDAs might lead to a new type of antiviral possibly helping to overcome existing problems with conventional anti-HCMV drugs. PROTACs are hetero-bifunctional molecules containing two elements joined together by a linker, i.e., firstly, a target-binding molecule, and secondly, an e E3 ligase-recruiting unit that, by polyubiquitination induction, drives the target protein into proteasomal degradation. Our investigation of THAL-SNS032, a commercially available CDK9-directed PROTAC, provided first evidence that this strategy can yield drugs with a strong antiviral efficacy. Here, THAL-SNS032 revealed concentration-dependent anti-HCMV activity in a non-cytotoxic range. The antiviral activity coincided with the mid-nanomolar concentration range necessary for the drug-induced target degradation. Moreover, this PROTAC-CDK9 exerted some broadness of antiviral activity, including strains of HCMV, murine CMV, and even SARS-CoV-2, while other human viruses were not susceptible. On this basis, the antiviral potential of PROTAC-based experimental drugs is discussed, including questions of drug efficacy, mode of activity, and combination treatment options.

## 2. Results and Discussion

### 2.1. Assessment of PROTAC-Mediated Degradation of Target Proteins 

**Figure 1:**
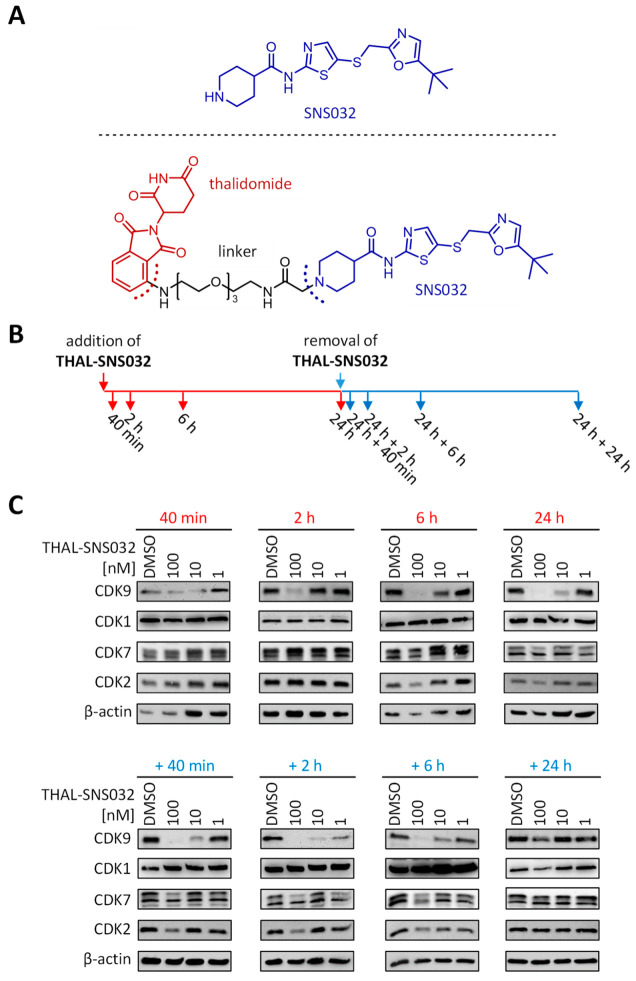
THAL-SNS032 induces degradation of CDKs in HFFs. (**A**) Chemical structures of SNS032 (upper panel) and THAL-SNS032 (lower panel). (**B**) Parallel cultures of HFFs were treated with 100, 10, or 1 nM of THAL-SNS032 and cells were harvested at 40 min, 2, 6, and 24 h. After 24 h of treatment, a removal and washout of THAL-SNS032 was performed and cells were then harvested at additional 40 min, 2, 6, and 24 h. (**C**) Cell lysates were used in Western blot analyses to detect the indicated CDKs; β-actin served as loading control.

Although PROTACs are generally considered to possess superior biological activities, their effects, including target protein degradation, have mostly been studied on in vitro cancer models. The vast majority of these PROTACs are derived from protein kinase inhibitors (PKIs). THAL-SNS032 (which primarily binds and, thereby, induces degradation of CDK9, but also of CDKs 1, 2, and 7, albeit at higher concentrations) is the PROTAC that was obtained by linking the E3-recruiting unit thalidomide to the PKI SNS032 (Figure 1A). Given the strong dependence of HCMV replication on host cell CDKs, THAL-SNS032 has been a promising candidate for a potential prototypic anti-HCMV PROTAC. To analyze whether THAL-SNS032 induces degradation of its target proteins in HFFs and to determine the kinetics of CDK depletion, cells were treated with increasing drug concentrations along a course of consecutive time points (Figure 1B). After 24 h of treatment, a drug removal and washout was performed to follow the reconstitution of CDK levels by sample collections during the respective time intervals. For all time points, total cellular lysates were prepared and levels of the primary target CDK9, as well as the secondary targets CDK1, 2, and 7, were determined by Western blot analysis (Figure 1C). Notably, CDK9 was most responsive to THAL-SNS032 at an applied 100 nM concentration, revealing decreased protein levels after 40 min. Beginning with 6 h—10 nM of THAL-SNS032 markedly reduced the CDK9 level. In addition, CDK2 and the larger variety of CDK7 revealed a decline in protein levels when treated with 100 nM THAL-SNS032 for 6 to 24 h. In contrast to CDKs 2, 7, and 9, CDK1 was apparently not affected by THAL-SNS032 treatment. After the washout step, reduced levels of CDK9, 2, and 7 were maintained for 6 h, while all CDKs reconstituted their initial protein levels after 24 h in the absence of the drug.

**Figure 2:**
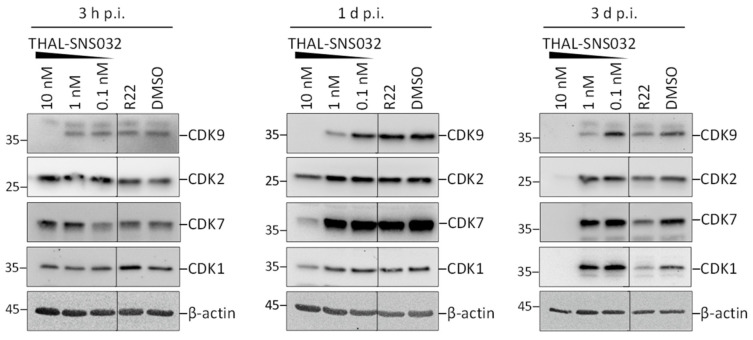
THAL-SNS032 induces degradation of CDKs in HCMV-infected HFFs. HFFs were inoculated with HCMV-AD169 at a multiplicity of infection (MOI) of 2, and treated with THAL-SNS032 or controls (CDK9 inhibitor R22 [1 µM], solvent control DMSO) immediately after infection. After 3 h, 1 day or 3 days, cells were harvested and subsequently analyzed for their CDK steady-state levels by Western blot stainings; β-actin served as loading control.

In general, HCMV induces upregulated or downregulated expression of several CDK-cyclin complexes, respectively, to build an intracellular environment, termed pseudomitosis, which promotes viral replication [32]. In this context, the addition of a CDK-targeting PROTAC may have striking consequences, theoretically ranging from a PROTAC-mediated abrogation of virus-induced CDK upregulation, associated with virus inhibition, to some moderate level of PROTAC resistance of HCMV-infected cells, based on the abundantly upregulated CDK pools. To address this question, an investigation of the THAL-SNS032-induced CDK degradation in HCMV-infected HFFs was performed in a similar Western blot-based kinetic experiment. Interestingly, with HCMV-infected HFFs, a reduction of CDK levels was observed at a lower concentration of THAL-SNS032 than that seen for uninfected HFFs. In infected cells, a concentration of 10 nM THAL-SNS032 was sufficient to deplete CDK9 levels at 3 h post-infection (h p.i.) and a very low concentration of 1 nM still imposed a partial reduction on CDK9 levels at 1 and 3 d p.i. (Figure 2). The levels of the three other CDKs monitored in this approach were markedly reduced at 1 d p.i. and completely lost at 3 d p.i. Notably, this reduction also applied for CDK2, which, in the previous setting, was completely inert towards degradation (Figure 2). During the 3-d period of experimental duration, representing one round of HCMV replication, a sustained degree of CDK suppression was observed, thereby demonstrating that the PROTAC effect completely counteracted the HCMV-driven CDK upregulation. As a non-CDK-degrading reference compound, the effect of R22, a specific CDK9 inhibitor with previously reported anti-HCMV activity, was analyzed in parallel [28]. As expected, R22 did not affect the levels of CDKs at 3 h or 1 d p.i., whereas at 3 d p.i., a slight reduction of CDKs 2, 7, and 9 was observed, in addition to a more pronounced effect seen for CDK1. This phenomenon represents a likely consequence of the anti-HCMV activity of R22, which in turn antagonizes the upregulation of CDKs by HCMV, an effect that became apparent in this setting at a later stage of the viral replication cycle. Since THAL-SNS032 affects the protein levels of its primary target CDK9 already after 3 h p.i., the PROTAC strategy seems to be exceptionally potent in interrupting the mutual feedback loop between HCVM-driven upregulation of CDKs and the CDK-mediated support of HCMV replication.

### 2.2. THAL-SNS032 and SNS032 Inhibit HCMV Replication in Primary Human Fibroblasts 

**Figure 3:**
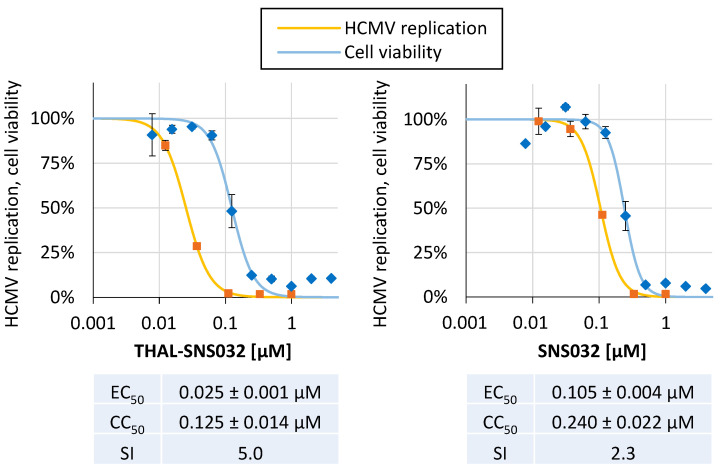
Comparative analysis of the anti-HCMV activity exerted by THAL-SNS032 and SNS032. THAL-SNS032 and SNS032 were assayed for their antiviral activity in a GFP-based replication assay using HCMV AD169-GFP for the infection of HFFs. Antiviral compounds were added immediately p.i., starting at 1 µM followed by five three-fold dilution steps. Cells were lysed; 7 d p.i. to perform quantitative GFP fluorometry. Cell viability was determined by performing NRA in parallel uninfected cells. Values represent mean ± SD of triplicate (NRA) or quadruplicate (GFP) determinations. One representative experiment of at least four independent replicates is depicted.

To evaluate the anti-HCMV activity of THAL-SNS032 and the parental, non-PROTAC compound SNS032, both compounds were tested in a reporter-based HCMV multi-round replication assay using a green fluorescence protein (GFP)-expressing recombinant virus. Antiviral activity was defined as treatment-induced reduction of the GFP signal compared to solvent-treated cells. On this basis, the effective concentrations required for half-maximal reduction of viral replication (EC_50_) were calculated (Figure 3). For the in vitro anti-HCMV activity of THAL-SNS032, an EC_50_ value of 0.025 ± 0.001 µM was determined, which was reduced four-fold compared to SNS032 with 0.105 ± 0.004 µM. Noteworthy, these mid-nanomolar concentrations of THAL-SNS032 required for inhibition of viral replication coincided with those required for degradation of all target CDKs in HCMV-infected HFFs (Figure 2). This finding suggested that, in addition to the primary target CDK9, the degradation of at least one further CDK may have been responsible for antiviral activity. Cytotoxicity was determined in parallel by the Neutral Red uptake assay (NRA), using uninfected HFFs treated for the respective periods with either of the compounds. The CC_50_ values for THAL-SNS032 and SNS032 were 0.125 ± 0.014 µM or 0.240 ± 0.022 µM, respectively. Importantly, for THAL-SNS032, concentrations lower than 0.1 µM induced suppression of viral replication in the absence of detectable cytotoxicity. In comparison, SNS032 showed a considerable overlap in concentrations of antiviral and cytotoxic activity (Figure 3). This characteristic is also reflected by the SI values of 5.0 and 2.3 for THAL-SNS032 and SNS032, respectively.

### 2.3. Analysis of the THAL-SNS032 Drug Interaction with the CDK7 Inhibitor LDC4297 or with the CDK8 Inhibitor SEL 120 Using the Loewe Additivity Fixed-Dose Assay

**Figure 4:**
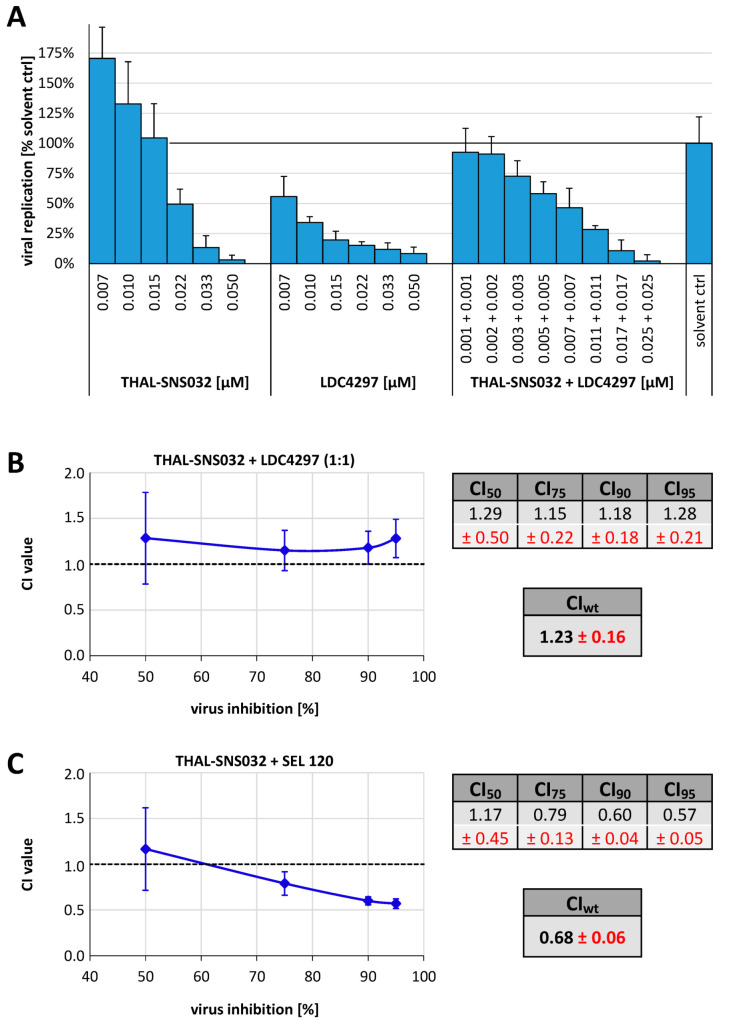
Analysis of the THAL-SNS032 drug interaction with LDC4297 using the Loewe additivity fixed-dose assay. (**A**) HCMV AD169-GFP-infected HFFs are treated with the indicated concentrations of THAL-SNS032 and LDC4297, either as a single-compound treatment or as a combination of both. Inhibition of viral replication was determined by quantitating the cell-associated GFP fluorescence of compound-treated cells relative to the solvent control (DMSO). (**B**) The generated data were used to determine CI values at 50%, 75%, 90%, and 95% of virus inhibition. Subsequently, the weighted CI value (CI_wt_) was calculated as: (1 × CI_50_ + 2 × CI_75_ + 3 × CI_90_ + 4 × CI_95_)/10. (**C**) The drug interaction between THAL-SNS032 and the CDK8 inhibitor SEL120 was analogously determined and the respective CI and CI_wt_ values were calculated. Data represent means ± SD of three experimental replicates.

Several inhibitors of protein kinases, especially CDKs, have exerted strong anti-HCMV activity in vitro [22,23,25,26,29,31]. In particular, the CDK7 inhibitor LDC4297 displayed potent anti-CMV activity in vitro as well as in vivo [22,26]. Thus, we hypothesized that simultaneous inhibition of CDK9 and CD7 will potentially exert exceptionally potent antiviral activity and, thus, reduce the effective doses and, consequently, mitigate cytotoxicity. To formally address this combinatorial antiviral drug interaction in vitro, the Loewe additivity was applied. Based on the preceding determination of the EC_50_ value for LDC4297 of 0.03 µM [22,26], which closely resembles the EC_50_ of THAL-SNS032, HCMV-infected HFFs were treated with identically concentrations of the single compounds centered on the respective EC_50_ values as well as a equimolar combination of both compounds (Figure 4A). The resulting raw data were analyzed using CompuSyn software (Version 1.0 [33]) based on the procedure developed by Chou and Talalay (1984, [34]). By this computational evaluation, the dose–effect curves for each drug or drug combination are converted to median effect plots, before CI values for the combinations are determined for 50% (CI_50_), 75% (CI_75_), 90% (CI_90_), and 95% (CI_95_) virus inhibition (Figure 4B). Here, a CI value of 1 implies additive interaction, <1 synergistic and >1 antagonistic. A weighted CI (CI_wt_) was calculated from the four aforementioned CI values, which uses a higher weighting for the desired strong inhibition. Synergy, antagonism, or additivity indicated by the CI_wt_ were defined as follows: values <0.1 to 0.3, strongly synergistic; 0.3 to 0.7, synergistic; 0.7 to 0.85, moderately synergistic; 0.85 to 0.9, slightly synergistic; 0.90 to 1.10, (nearly) additive; 1.10 to 1.20, slightly antagonistic; 1.20 to 1.45, moderately antagonistic; 1.45 to 3.3, antagonistic; 3.3 to >10, strongly antagonistic. For the THAL-SNS032 and LDC4297 combination treatment, the CI_50–90_ values were centered at 1.2 indicating a slight to moderate antagonism. Consequently, the CI_wt_ of 1.23 revealed an overall moderate antagonistic drug interaction. Although this result was unanticipated, it still can mechanistically be explained by the fact that, besides CDK9, THAL-SNS032 also degrades CDK7, the target of LDC4297, and thereby basically abrogates the antiviral effect of LDC4297. To address this issue in the absence of unwarranted antagonistic interference, the interaction of THAL-SNS032 with an inhibitor that targets CDKs outside the spectrum of SNS032 was additionally investigated. We employed the novel CDK8/19-specific PKI SEL120, which is currently being developed for treatment of acute myeloid leukemia [35], and so far has not been analyzed for anti-HCMV activity in vitro. Here, we specifically addressed the question of a putatively reinforcing interaction with THAL-SNS032 (Figure 4C). Since SEL120 revealed an EC_50_ value of 0.12 ± 0.07 µM, which was considerably higher than the EC_50_ of THAL-SNS032, ratios of 1:10 and 1:20 for THAL-SNS032 to SEL120 were analyzed. In contrast to LDC4297, SEL120 actually demonstrated synergistic potential in combination with THAL-SNS032, particularly at concentrations inducing at least 75% inhibition of viral replication, a finding that was consistent for all tested combination ratios. This synergistic interaction is also reflected by the corresponding CI_wt_ of 0.68, indicating a moderate level of drug synergism. 

### 2.4. THAL-SNS032 Inhibits HCMV Replication across Diverse Virus Strains and Cell Types

**Figure 5:**
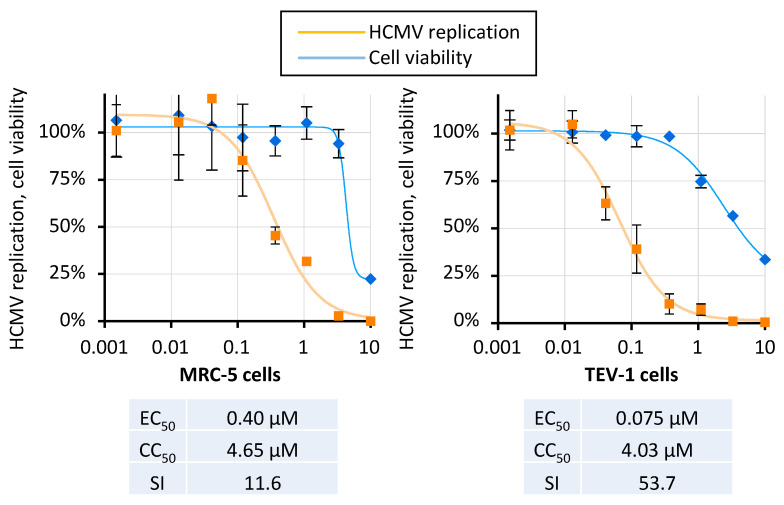
THAL-SNS032 inhibits HCMV replication in MRC-5 cells and human first-trimester extravillous trophoblast (TEV-1) cells. Merlin-infected MRC-2 and TEV-1 cells were with various concentrations of THAL-SNS032. Viral replication was quantitated by HCMV-specific qPCR using cell culture supernatants collected at 7 p.i. Cell viability was determined in parallel by a Neutral Red uptake assay using uninfected MRC-5 and TEV-1 cells.

To further corroborate the anti-HCMV activity of THAL-SNS032, additional replication systems using two susceptible human cell lines infected with the genetically intact HCMV Merlin strain were employed. As a first replication model, we used MRC-5 cells inoculated with HCMV Merlin at a multiplicity of infection (MOI) of 0.02. Following infection, cells were subsequently treated with THAL-SNS032 and viral replication was assessed 7 d p.i. qPCR quantitation of released viral genomes. In this setting, THAL-SNS032 reduced HCMV replication with an EC_50_ value of 0.04 µM (Figure 5). The corresponding analysis of cytotoxicity in MRC-5 cells revealed a CC_50_ value of 4.65 µM with 11.6 as corresponding SI. Additional combinations of MOIs 0.2, 0.02, or 0.002 with replication periods of 4 to 10 days were analyzed (Figure S1). In these settings, the lower MOIs of 0.02 and 0.002 resulted in submicromolar EC_50_ values ranged from 0.34 to 0.94 µM, whereas an MOI of 0.2 led to increased EC_50_ values of 1.77 and 3.07 µM when determining 4 or 7 d p.i., respectively. As another virus-permissive system, the human first-trimester extravillous trophoblast cell line (TEV-1) was infected with HCMV, treated with THAL-SNS032, and viral replication was followed for 7 d. Quantitation of replication by HCMV-specific qPCR revealed a dose-dependent viral inhibition with a resulting EC_50_ of 0.075 µM (Figure 5), which was comparable to the value determined for HFF (Figure 3). Cell viability determined in parallel resulted in a CC_50_ value of 4.03 µM and, consequently, an SI of 53.7. Moreover, the concentration that achieved 90% inhibition of viral replication (EC_90_) did not impact cell viability (Figure S2, GCV used as a reference control drug). It is noteworthy to mention that, particularly when comparing this HCMV Merlin/TEV-1 system to the HCMV AD169-GFP/HFF system, antiviral activity was observed in a similar concentration range. However, the respective CC_50_ concentrations substantially differed, demonstrating that the cytotoxicity induction is strongly dependent on the cell type used. Moreover, transwell migration assays were performed to investigate the effects of THAL-SNS032 treatment in resolving HCMV-induced inhibition of trophoblast migration (Figure S3). As we have previously demonstrated, HCMV AD169 infection significantly inhibited trophoblast migration compared to mock-infected cells (*p* < 0.0001). Treating cells with 10 µM ganciclovir (GCV) showed no effect in resolving HCMV inhibition of migration compared with infected untreated cells (*p* = 0.999). Treating cells with 0.5 µM THAL-SNS032 almost completely abolished trophoblast migration consistent with the importance of CDK9 in cell migration [36,37,38]. Immunofluorescence analysis showed that there was no significant difference in levels of the HCMV immediate early protein expression between the treatment groups 24 h p.i. (data not shown).

### 2.5. THAL-SNS032 and SNS032 Reveal Comparable Activities against Murine CMV In Vitro

**Figure 6:**
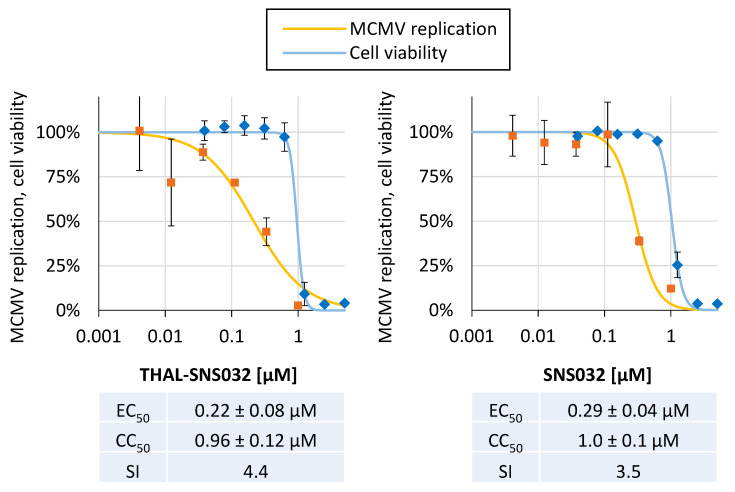
Comparable analysis of anti-MCMV activity of THAL-SNS032 and SNS032. Bot compounds were analyzed for their antiviral activity in a GFP-based replication assay by titration on MCMV Smith-GFP-infected MEFs. A concentration of 1 µM was used as the starting point followed by six three-fold dilution steps. The cell-associated GFP fluorescence was used to quantitate viral replication 5 d p.i. Cell viability was determined in parallel using uninfected MEFs incubated with a compound for 5 d. Values represent means ± SD of biological triplicates (NRA) or quadruplicates (GFP).

Next, we investigated whether the anti-cytomegaloviral activity of THAL-SNS032 and SNS032 was conserved between human and animal CMVs. For this purpose, the antiviral activity against the murine CMV (MCMV) was analyzed in mouse embryonic fibroblasts (MEFs). Titration of THAL-SNS032 and SNS032 exerted a dose-dependent reduction on MCMV replication (Figure 6). EC_50_ values for THAL-SNS032 and SNS032 were determined as 0.22 ± 0.08 µM or 0.29 ± 0.48 µM, respectively. The corresponding CC_50_ values were in the range around 1 µM for both compounds. It appears noteworthy that these EC_50_ and CC_50_ values were higher when compared to HCMV-infected HFFs, possibly due to lower binding affinities of THAL-SNS032 and SNS032 towards murine CDKs compared to human CDKs. Additionally, the biological activities of these two compounds, with or without PROTAC moiety, displayed no noticeable difference in this replication model. This is most likely explained by the fact that, contrary to the human CRBN, which serves as the receptor for thalidomide-related PROTAC moieties, its murine homolog fails to engage in this interaction [39,40]. Consequently, in context of murine host cells, the THAL-SNS032 obviously acts, like SNS032, as a plain CDK9 inhibitor without the additional benefit of target degradation.

### 2.6. THAL-SNS032 and SNS032 Potently Inhibit SARS-CoV-2 Replication in a Human Cell Line

**Figure 7:**
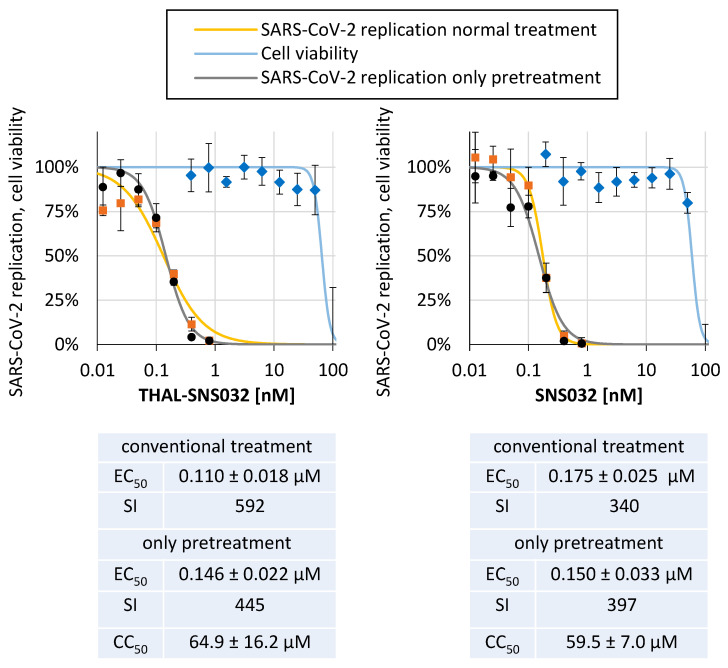
Anti-SARS-CoV-2 activity of THAL-SNS032 and SNS032 in a permissive human cell line. Caco-2 cells were cultivated in 96-well plates at 25,000 cells/well, infected with the recombinant SARS-CoV-2 d6-YFP reporter virus at an MOI of 0.003 and treated with the indicated concentrations of THAL-SNS032 and SNS032. At 30 h p.i., cells were harvested and viral replication was determined by quantitative fluorescence detection of virus-driven YFP expression using the fixed cells. Cell viability was determined from parallel cultures of uninfected Caco-2 cells by determination of release LDH after 48 h of treatment. Values represent means ± SD of biological quadruplicates (replication) or triplicates (LDH release). One representative experiment out of three is depicted.

We previously demonstrated that CDK inhibitors impair SARS-CoV-2 replication in human Caco-2 cells [41]. The effective compounds were comprised by the CDK1/2/5 inhibitor R25/alsterpaullone and SNS032, whereas the CDK7 inhibitor LDC4297 was inactive. Since the SNS032 exerted antiviral activity in this in vitro model in absence of cytotoxic effects, this system seemed particularly suited for a side-by-side comparison of THAL-SNS032 and SNS032. To this end, Caco-2 cells were infected with the YFP-expressing recombinant SARS-CoV-2 d6-YFP and treatment with THAL-SNS032 or SNS032 started concomitantly with virus addition. Quantitation of viral replication by measurement of the cell-associated YFP fluorescence 30 h p.i. revealed a dose-dependent inhibition with EC_50_ values of 0.110 ± 0.018 µM and 0.175 ± 0.25 µM for THAL-SNS032 and SNS032, respectively (Figure 7). Levels of cytotoxic concentrations were comparable for both compounds in Caco-2 cells, resulting in a slightly more favorable SI for THAL-SNS032 compared to SNS032. To better capitalize on the CDK-degrading activity of THAL-SNS032 for the antiviral activity, cells were 24 h pretreated with THAL-SNS032 or SNS032 and the infection and subsequent viral replication cycles were performed in the absence of any compound. Since the CDK9 levels in HFFs remained reduced even after withdrawal of THAL-SNS032 (Figure 1 and Figure 2), we hypothesized whether, in the context of pretreatment with the subsequent compound washout, THAL-SNS032 might have a sustained antiviral effect, whereas the SNS032-mediated inhibition might possibly be reversed. Surprisingly, however, not only were the antiviral activities of THAL-SNS032 and SNS032 found to be virtually indistinguishable, but also the dose–response in this pretreatment setting was very comparable to the conventional treatment scheme (Figure 7). A mean of three experimental replicates revealed EC_50_ ratios of 1.6 (conventional treatment) and 1.7 (pretreatment), respectively, when setting SNS032 in relation to THAL-SNS032 (Table 1). Thus, a tendency of lower EC_50_ values and more favorable SI values was noted for THAL-SNS032 compared to SNS032.

### 2.7. Conclusions: THAL-SNS032 Signifies the High Potential of a New Type of PROTAC-Based Antiviral Drugs

In the current study, we analyzed the in vitro activity of THAL-SNS032 against various viruses and additionally compared THAL-SNS032 to its non-PROTAC parental drug SNS032 (Table 1). THAL-SNS032 revealed antiviral activity for several DNA and RNA viruses, while other viruses behaved insensitive. Even comparing the three analyzed herpesviruses, including viral strains, only the CMVs were found sensitive to THAL-SNS032 treatment, but varicella zoster virus (VZV) remained unaffected. For HCMV, EC_50_ values ranged from 0.03 to 3.07 µM and this two-log stretch seems to strongly depend on the cell type and the viral MOI used for infection. Similarly, the CC_50_ values differed substantially between these analyzed primary cells and immortalized cell lines. This kind of variation appears plausible on the basis that the cellular target CDKs, as well as the E3 ligase CRBN, might possess cell type-dependent expression levels. Consequently, SI values for CMVs ranged from 5 to 13 in the viral replication systems analyzed, demonstrating that THAL-SNS032 has indeed a true anti-cytomegaloviral activity that is separable from the range of cytotoxicity. When comparing the anti-HCMV effect of THAL-SNS032 to its non-PROTAC counterpart SNS032, the THAL-SNS032 exerted a 3.7-fold stronger antiviral activity. This illustrates that this prototypic CDK9-targeted PROTAC, which exerts the intended degradative mechanism as experimentally verified, presents a measurable advantage over the non-PROTAC parental inhibitor. Notable, a similarly sized five-fold increase in activity was recently described for anti-coronaviral PROTACs based on indomethacin [42], indicating that this magnitude is easily achievable by converting an inhibitor to its corresponding PROTAC, an approach that is still open for optimization as a focus of our continued studies on antiviral options against human pathogenic viruses, including HCMV. 

Concerning MCMV that represents an animal model candidate virus, the picture was quantitatively different to HCMV, since both drugs revealed comparable EC_50_ and CC_50_ values; thus, no evidence for the superiority of the PROTAC approach was given in this case. This appeared not unexpected, since thalidomide does not, or only poorly, binds murine CRBN, and for this reason can neither efficiently induce target ubiquitination nor degradation [39,40]. Consequently, although thalidomide as a building block for the synthesis of PROTACs is cheap and accessible, thalidomide-based PPOTACs may not be properly be evaluated in vivo using a murine/MCMV replication model. Concerning another human cell-based analysis system, the inhibitory potential of THAL-SNS032 onto SARS-CoV-2 replication in Caco-2 was analyzed. Since Caco-2 cells tolerate much higher concentrations of both THAL-SNS032 and SNS032, this system appeared particularly suited for a comparative characterization of antiviral activity. Surprisingly, both compounds revealed very similar antiviral activities, with only moderately increased efficacy of THAL-SNS032 versus SNS032, and this result was similarly obtained for conditions without or with drug pretreatment of cells (Table 1). Thus, further PROTAC-specific analysis of SARS-CoV-2 is required to illustrate this issue. 

Additional DNA and RNA viruses were included, so that the effect of THAL-SNS032 towards Zika virus (ZIKV) replication in Vero cells was investigated, and here, surprisingly, even concentrations up to 10 µM did not affect virus replication in our system, although inhibition of ZIKV replication in the human astrocytoma cell line SNB-19 using micromolar concentrations of CDK inhibitors was reported elsewhere [43]. Moreover, an anti-adenoviral effect has been described for the CDK9 inhibitor FIT039 [44]. Thus, we investigated THAL-SNS032 and SNS032 on HFFs infected with human adenovirus type 2 (HAdV-2), but none of the drugs produced a measurable impact on the HAdV-2 plaque formation assay, at least at concentrations (0.03 µM) that in parallel produced anti-HCMV activity. 

In comparison, the use of PROTACs in cancer models have shown major beneficial features, which might be promising for broader use, including antiviral therapy. In this field, evidence has been provided that PROTACs induce specific target degradation and, thus, act more efficiently as pharmaceutical inhibitors than their non-PROTAC counterparts [45,46,47]. Moreover, by converting conventional enzymatic inhibitors to PROTACs, even enzyme mutants, exhibiting resistance to the initial drug, were subject to degradation [48,49,50,51,52]. Concerning novel strategies of antiviral targeting, such benefits might likewise be provided for direct-acting antivirals as well as host-directed antivirals. PROTACs, based on their specific resistance-reducing qualities, represent a valuable option to deal with the rapid evasion of viruses driven by low-fidelity polymerases, large numbers of progeny viruses originating from one single infected cell, and short-term replication cycles. In addition, the potential of PROTACs is not restricted to enzymes, but can also be applied to non-enzymatic target proteins, i.e., by identification of small molecule ligands binding viral proteins, new PROTACs may be generated to extend the drug-accessible range of virus-encoded targets. Through the PROTAC-mediated degradation of viral proteins, either possessing regulatory or structural functions, previously inaccessible key steps of viral replication cycles may be exploited. Most importantly, PROTACs have the ability to degrade their targets in various subsequent cycles (substoichiometric mode of action) that are not restricted to the primary target, but might also involve binding partners in the context of multiprotein complexes [53,54]. As a direct consequence, highly ordered assemblies of virion structures composed of multiple copies of viral structural proteins may be subject to drug-targeted disruption. For this reason, our prioritized approach with currently synthesized PROTACs will be directed to multiprotein complexes essential for virus replication. Despite these potential benefits, only telaprevir-based PROTACs active against the hepatitis C virus and indomethacin-derived PROTACs with anti-coronavirus activity have been reported on so far [42,55]. Taken together, data of this study underline that PROTACs harbor untapped, possible game-changing potential in regard to antiviral drug development, so that skillful procedures of PROTAC synthesis and a structure–activity relationship may guarantee further success in the near future.

## 3. Materials and Methods 

### 3.1. Cells and Viruses

Primary human foreskin fibroblasts (HFFs, derived from clinical samples, Children’s Hospital, Erlangen, Germany) were grown in Eagle’s Minimal Essential medium (MEM) supplemented with 1× GlutaMAX^TM^ (both Thermo Fisher Scientific, Waltham, MA, USA), 10 μg/mL gentamicin and 10% fetal bovine serum (FBS, Capricorn, Ebsdorfergrund, Germany). Mouse embryonic fibroblasts (MEFs, ATCC, Manassas, VA, USA) were cultivated in Dulbecco’s Modified Eagle Medium (DMEM, Thermo Fisher Scientific), supplemented with 1× GlutaMAX^TM^, 10 μg/mL gentamicin and 10% FBS. Human Caco-2 cells were cultivated at 37 °C, 5% CO_2_ and 80% humidity using Dulbecco’s Modified Eagle Medium (DMEM, 11960044, Thermo Fisher Scientific, Waltham, MA, USA) supplemented with 2 mM GlutaMAX^TM^ (35050038, Thermo Fisher Scientific), 10 µg/mL gentamycin (22185.03, SERVA, Heidelberg, Germany), 10% FBS and 1% MEM Non-Essential Amino Acids Solution (11140050, Thermo Fisher Scientific). Vero 76 were maintained in DMEM containing 1× GlutaMAX^TM^, 10 µg/mL gentamycin and 10% FBS. Primary human lung fibroblasts (MRC-5 cells) were maintained in MEM media, supplemented with 100 U/mL penicillin G, 100 µg/mL streptomycin and 29.2 µg/mL L-glutamine (1× PSG) and 10% FBS. Vero cells for Zika virus investigations were maintained in DMEM/F12+Gluta-MAX, 1× PSG and 10% FBS. TEV-1 cells were maintained in Ham’s F10 Nutrient mix, 1× PSG and 10% FBS. All cultured cells were maintained at 37 °C, 5% CO_2_ and 80% humidity and regularly monitored for absence of mycoplasma contamination (Lonza™ MycoAlert™, Thermo Fisher Scientific, Waltham, MA, USA). Recombinant HCMV strain AD169 expressing green fluorescent protein (AD169-GFP, [56]), and recombinant MCMV strain Smith (MCMV-GFP) were used for in vitro replication assays in HFFs or MEFs, respectively. Genetically intact HCMV strain Merlin (UL128+, RL13−) was derived from a Merlin-BAC recombinant, pAL1120, and propagated in RPE-1 cells, as previously reported [57]. The Asian lineage of Zika virus (PRVABC59) was propagated in Vero cells and plaque assays in Vero cells used to assess viral titer. Recombinant varicella zoster virus strain Oka (VZV-GFP) was generated by the use of bacmid technology, as described elsewhere [58], and virus propagation, including infection experiments for antiviral testing based on GFP fluorometry, was performed in a cell-associated manner on HFFs under standard conditions [26,59,60]. The generation and characterization of the recombinant SARS-CoV-2 virus d6-YFP in which viral ORF6 was replaced with EYFP has been described previously [41,61]. All SARS-CoV-2 infection experiments were performed under BSL-3 conditions.

### 3.2. Antiviral Compounds

Stock aliquots of THAL-SNS032 and SNS032 (both Tocris, Wiesbaden-Nordenstadt, Germany), SEL 120 (MedChemExpress, Monmouth Junction, NJ, USA), roscovitine (Millipore-Calbiochem) and R22 (GPC Biotech AG, Martinsried, Germany) were prepared in DMSO and stored at −20 °C.

### 3.3. Assays for Determination of Antiviral Activity of Test Compounds

The GFP-based HCMV replication assay was performed as described previously [26,56]. Briefly, parallel cultures of HFFs were inoculated with a virus stock dilution resulting in 25% GFP-positive cells at 7 d p.i. (i.e., 1× TCID_25_^7d^). Viral inocula were replaced after 90 min with cell culture medium containing desired concentrations of test compounds. Antiviral activities were quantitatively determined as reduction of GFP fluorescence in cell lysates relative to the DMSO control 7 d p.i. Determination of anti-MCMV efficacy was based on a GFP-based approach similar to the HFF/HCMV setting by using MCMV-GFP to infect MEFs. MEFs seeded at 380,000 cells/well in 6-well plates the day before infection were inoculated with MCMV stock for 90 min and immediately treated after infection. Moreover, 5 d p.i. cells were lysed and viral replication was quantitated by automated fluorometry. For determination of anti-adenoviral activity, HFF 200,000 cells were seeded in 12-well plates the day before infection. Cells were inoculated with HAdV-2 for 90 min, before replacing viral inocula with MEM-agarose mixtures containing test compounds. After 10 days, agarose overlays were removed and plaque was detected by staining cell layers with crystal violet (1% dissolved in 20% ethanol), followed by several washing steps with PBS and, subsequently, counting under a microscope. The SARS-CoV-2 replication assay using human Caco-2 cells was performed as described earlier [41]. Briefly, Caco-2 cells were infected with the recombinant SARS-CoV-2 d6-YFP reporter strain at an MOI of 0.003 in the presence of test compounds. At 30 h p.i., cells were fixed with 10% formalin and viral replication was assessed by YFP quantitation in a Victor X4 microplate reader (PerkinElmer, Waltham, MA, USA). Viral replication was assessed quantitation of the cell-associated YFP fluorescence and is presented as mean values of biological quadruplicates ± SD. All SARS-CoV-2 infection experiments were performed under BSL-3 conditions. Analysis of anti-HCMV activity by qPCR was performed as previously described [57]. Briefly, cells were seeded in 24-well plates and inoculated with virus in triplicate at specified multiplicities of infection (MOI). Plates were centrifuged at 770× *g* for 30 min followed by 2 h of incubation at 37 °C with 5% CO_2_. Supernatant was removed and replaced with fresh medium with or without antivirals and incubated at 37 °C. Total nucleic acid from cell culture supernatant was extracted and qPCR for viral genome copies was performed. For Zika virus quantification, qPCR was performed with ZIKV specific primers 5′-CTGTGGCATGAACCCAATAG-3′, 5′-ATCCCATAGTGCACCACTCC-3′, and probe 5′-(6FAM)CCACGCTCCAGCTGCAAAGG-3′. Reactions were carried out under the following conditions: reverse transcription at 55 °C for 20 min, denaturation at 95 °C for 3 min, followed by 45 cycles of denaturation at 94 °C for 15 s, hybridization and elongation at 60 °C for 1 min using Superscript III OneStep RT-PCR System (Thermo Fisher Scientific). Transwell migration assays were performed as previously described [62]. TEV-1 cells were infected with AD169 at an MOI of 2 in the presence of test compounds (THAL-SNS032 at 0.5 µM and ganciclovir at 10 µM). At 24 h p.i., cells were transferred into transwell inserts in the presence of test compounds with 200 ng/mL wnt-5a protein to facilitate cell migration and cells allowed to migrate for 21 h. Cells were then fixed, stained with 1% crystal violet and analyzed as previously described [62]. Aliquots of infected cells were seeded in 8-well chamber slides and stained for CMV IE protein as previously described [63].

### 3.4. Determination of Cell Viability by Neutral Red Uptake and Lactate Release Assay

Drug induced cytotoxicity in HFF and other cell types was measured as a reduction of cell viability determined by Neutral Red uptake assay (NRA), as described previously [64,65]. Briefly, compound treated cells were incubated with a final concentration of 40 μg/mL Neutral Red (Sigma Aldrich, St. Louis, MO, USA) for 1 to 4 h, depending on the cell type. The amount of incorporated Neutral Red was released from the cells by incubation with destaining solution (50% ethanol, 49% H_2_O, 1% acetic acid), and subsequently quantitated in a microplate reader by fluorescence measurement using 560/630 nm for excitation/emission, respectively. For determination of lactate dehydrogenase release into the cell culture supernatant, the CytoTox 96^®^ Non-Radioactive Cytotoxicity Assay (Promega, Madison, WI, USA) was used according to the manufacturer’s protocol. Values were normalized to maximal release of LDH by measuring lysates of solvent-treated control cells. Cell viability was defined as the absence of cytotoxicity. 

### 3.5. Western Blot and Antibodies

Western blot analyses of infected and mock-infected HFFs were performed as previously described [27,66]. Antibodies specific for CDK9 (sc-484, Santa Cruz Biotechnology, Dallas, TX, USA), mAb-CDK1 (sc-54, Santa Cruz Biotechnology), pAb CDK2 (Sc-163, Santa Cruz Biotechnology), pAb-CDK7 (sc-723, Santa Cruz Biotechnology), and mAb β-actin (A5441, Sigma Aldrich, St. Louis, MO, USA) were used with an appropriate secondary antibody to detect the respective proteins. 

### 3.6. Loewe Fixed-Dose Assay Adapted to HCMV-GFP In Vitro Infection

Loewe additivity analyses were performed to using the HCMV GFP-based replication assay, according to previously described protocols [26,56]. HFFs were seeded at 1.6 × 10^5^ cells per well in 12-well culture plates and infected the next day with 1× TCID_25_^7d^ (i.e., 25% GFP-forming dose of a multi-round infection measured at 7 d p.i.) of HCMV AD169-GFP [56]. After 90 min of virus adsorption, inocula were exchanged for new media containing test compounds, as a single compound, a compound combination, or solvent control. The highest concentrations were chosen to approximately match 4× EC_50_ of the respective compounds, and 6 (for single compounds) or 8 (for compound combination) subsequent serial 1.5-fold dilutions thereof were analyzed. All infections were performed in biological duplicates. Cells were lysed, 7 d p.i., and viral replication quantitated by fluorometry in the cell lysates in a Victor X4 microplate reader (PerkinElmer, Waltham, MA, USA) as mean of duplicate measurements of biological duplicates. Antiviral efficacy was expressed as the percentage reduction compared to the solvent control. Resulting values were subsequently analyzed for drug interaction using CompuSyn software (Version 1.0 [33]; ComboSyn, Inc., Paramus, NJ, USA). Results were considered as valid if all r values were above 0.90 and EC_50_ values closely matched previously determined concentrations. CI values extrapolated at 50, 75, 90, and 95% virus inhibition. 

## Figures and Tables

**Table 1:** Summary of antiviral activities in various virus replication models *.

					THAL-SNS032			SNS032			EC_50_ Ratio SNS032/ THAL-SNS032
Virus	Strain/ Type	MOI	d p.i.	Cell Type	EC_50_ [µM]	CC_50_ (µM)	SI	EC_50_ [µM]	CC_50_ (µM)	SI	
HCMV	AD169-GFP	1× TCID_25_^7d^	7	HFF	0.03 ± 0.01	0.18 ± 0.11	5.7	0.11 ± 0.02	0.22 ± 0.04	2.0	3.7
HCMV	Merlin		7	TEV-1	0.075	4.03	54	n.d.	n.d.	n.d.	n.d.
HCMV	Merlin	0.2	4	MRC-5	1.77	4.56	3	n.d.	n.d.	n.d.	n.d.
HCMV	Merlin	0.2	7	MRC-5	3.07	4.56	2	n.d.	n.d.	n.d.	n.d.
HCMV	Merlin	0.02	4	MRC-5	0.37	4.56	13	n.d.	n.d.	n.d.	n.d.
HCMV	Merlin	0.02	7	MRC-5	0.40	4.56	12	n.d.	n.d.	n.d.	n.d.
HCMV	Merlin	0.002	7	MRC-5	0.57	4.56	8	n.d.	n.d.	n.d.	n.d.
HCMV	Merlin	0.002	10	MRC-5	0.94	4.56	5	n.d.	n.d.	n.d.	n.d.
MCMV	Smith-GFP	1× TCID_25_^5d^	5	MEF	0.21 ± 0.09	1.00 ± 0.22	4.7	0.29 ± 0.05	1.03 ± 0.05	3.5	1.4
VZV	Oka-GFP	1× TCID_25_^7d^	6	HFF	inactive	0.18 ± 0.11	n.d.	inactive	0.22 ± 0.04	n.d.	n.d.
HAdV-2	type 2		10	HFF	inactive	n.d.	n.d.	inactive	n.d.	n.d.	n.d.
SARS-CoV-2	d6-YFP	0.003	1.25	Caco-2	0.11 ± 0.02	64.9 ± 16.2	592	0.18 ± 0.03	59.5 ± 7.0	340	1.6
SARS-CoV-2 (pretreated)	d6-YFP	0.003	1.25	Caco-2	0.15 ± 0.02	64.9 ± 16.2	444	0.15 ± 0.03	59.5 ± 7.0	397	1.7
ZIKV	PRVABC59	0.02	1	Vero	inactive	n.d.	n.d.	n.d.	n.d.	n.d.	n.d.
ZIKV	PRVABC59	0.02	2	Vero	inactive	n.d.	n.d.	n.d.	n.d.	n.d.	n.d.
ZIKV	PRVABC59	0.02	3	Vero	inactive	n.d.	n.d.	n.d.	n.d.	n.d.	n.d.
ZIKV	PRVABC59	0.02	4	Vero	inactive	43.9	n.d.	n.d.	n.d.	n.d.	n.d.

* n.d.; not determined.

## Supplementary Materials

The following are available online at https://www.mdpi.com/article/10.3390/ijms222312858/s1.
