# Supplementary material for: Development of a PROTAC-Based Targeting Strategy Provides a Mechanistically Unique Mode of Anti-Cytomegalovirus Activity
Source: Int J Mol Sci. 2021 Nov 27;22(23):12858. doi: 10.3390/ijms222312858 (PMC8657773; doi:10.3390/ijms222312858)
Supplement: Supplementary file 1 [file ijms-22-12858-s001.zip › ijms-1450229-supplementary.pdf]

Supplementary Material

Development of a PROTAC-based targeting strategy provides a mechanistically unique mode of anti-cytomegalovirus activity

Friedrich Hahn[1§], Stuart T. Hamilton[2], Christina Wangen[1], Markus Wild[1], Jintawee Kicuntod[1], Nadine Brückner[1], Jasmine E.L. Follett[2], Lars Herrmann[3], Ahmed Kheimar[4], Benedikt B. Kaufer[4], William D. Rawlinson[2], Svetlana B. Tsogoeva[3] and Manfred Marschall[1§]

[1] Institute for Clinical and Molecular Virology, Friedrich-Alexander University of Erlangen-Nürnberg (FAU), Erlangen, Germany; friedrich.hahn@uk-erlangen.de (F.H.), christina.wangen@uk-erlangen.de (C.W.), markus.wild@uk-erlangen.de (M.W.), jintawee.kicuntod@extern.uk-erlangen.de (J.K.), nadine.brueckner@fau.de (N.B.), manfred.marschall@fau.de (M.M.)

[2] Serology and Virology Division, NSW Health Pathology Microbiology, Prince of Wales Hospital, Schools of Women's and Children's Health, Medicine and Biotechnology and Biomolecular Sciences, University of New South Wales, Sydney, Australia; stuart.hamilton@health.nsw.gov.au (S.T.H.), j.follett@student.unsw.edu.au (J.E.L.F.), w.rawlinson@unsw.edu.au (W.D.R.)

[3] Institute of Organic Chemistry I, FAU, Erlangen, Germany; lars.herrmann@fau.de (L.H.), svetlana.tsogoeva@fau.de (S.B.T.)

[4] Institute of Virology, Freie Universität Berlin, Berlin, Germany; benedikt.kaufer@fu-berlin.de (B.B.K.), ahmed.kheimar@fu-berlin.de (A.K.)

Supplementary Figures

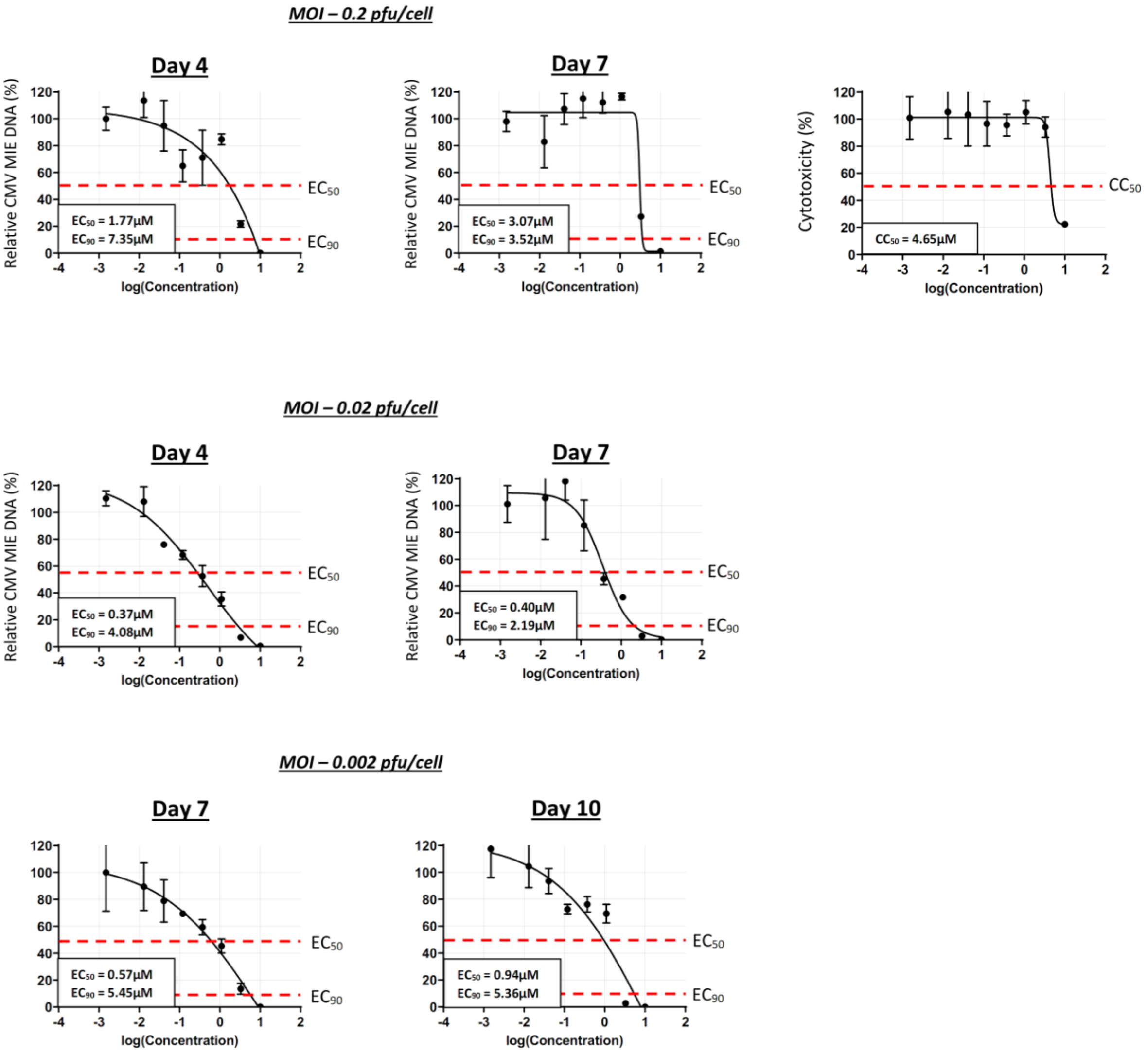

Figure S1. THAL-SNS032 inhibits HCMV replication in MRC-5 cells. HCMV MRC-5 cells were infected with HCMV Merlin at an MOI of 0.2, 0.02 or 0.002 and treated with various concentrations of THAL-SNS032. Viral replication was quantitated by HCMV-specific qPCR using cell culture supernatants collected at 4 to 10 d p.i., as indicated. Cell viability was determined in parallel by neutral red uptake assay using uninfected MRC-5 cells.

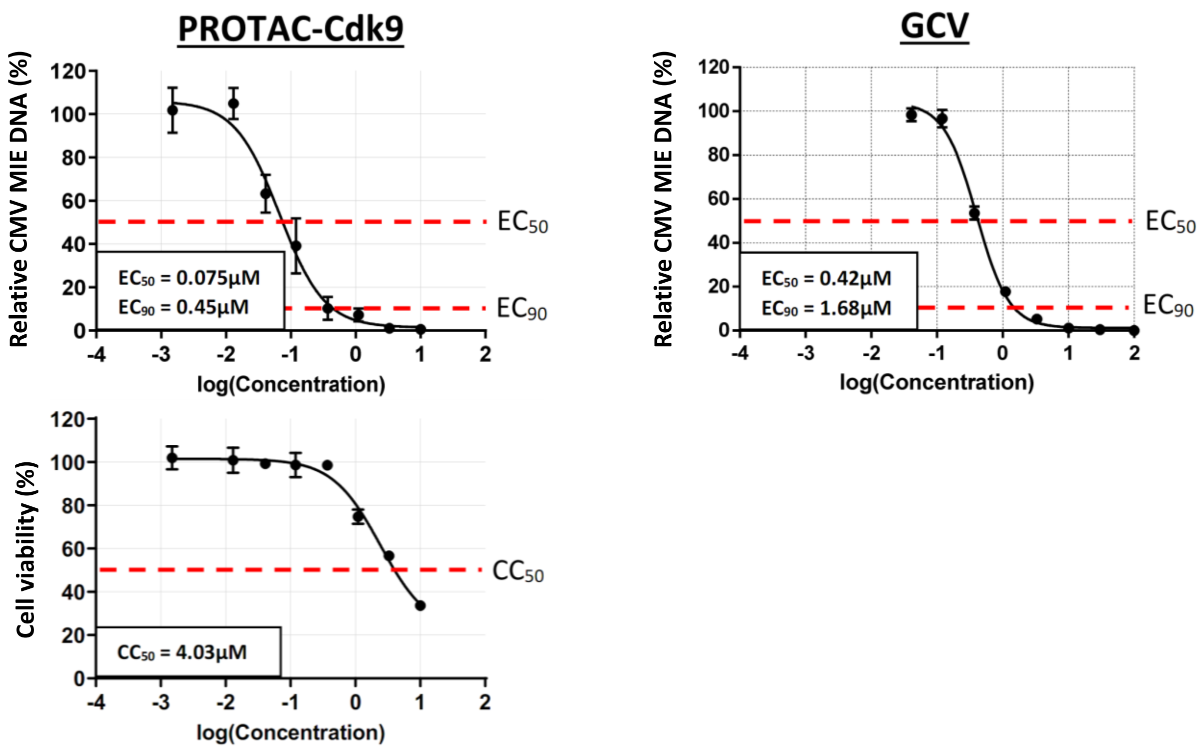

Figure S2. THAL-SNS032 inhibits HCMV replication in human first-trimester extravillous trophoblast cells (TEV-1). TEV-1 cells were infected with HCMV Merlin and THAL-SNS032 was added at various concentrations. Viral replication was quantitated by HCMV-specific qPCR using cell culture supernatants collected 7 d p.i.. Cell viability was determined in parallel by neutral red uptake assay using uninfected TEV-1 cells.

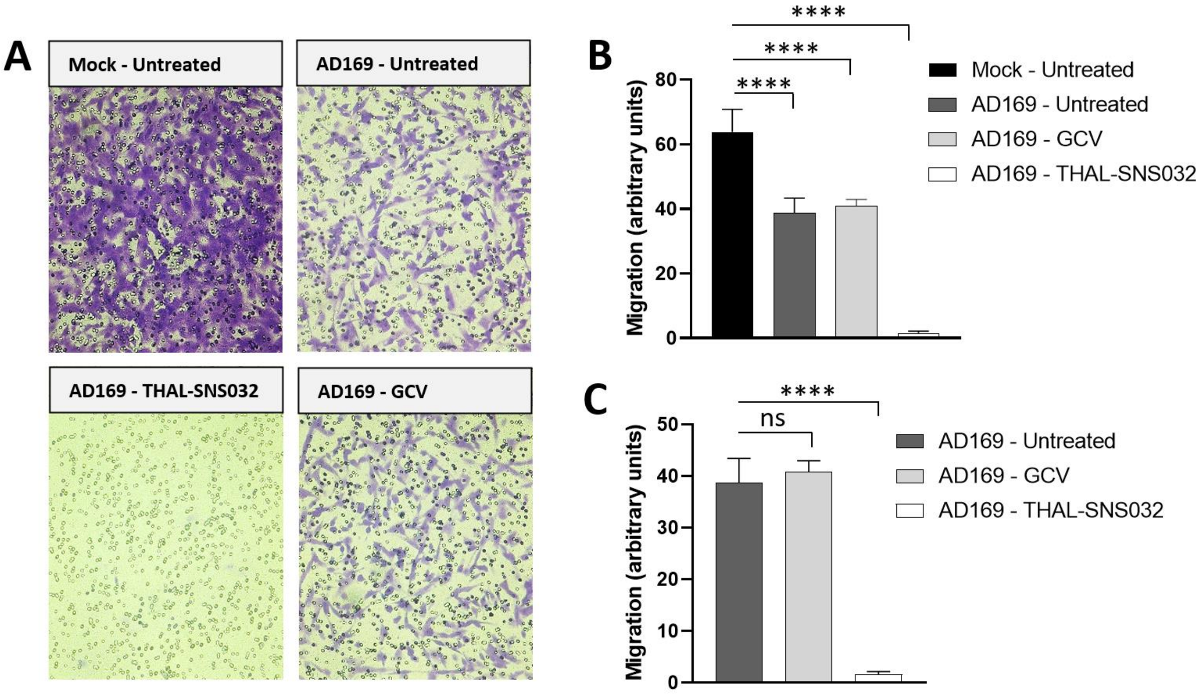

Figure S3. THAL-SNS032 inhibits cell migration in transwell migration assays. **(A)** TEV-1 trophoblast cells were infected with HCMV AD169 (MOI 2 pfu/cell) or mock-infected and the HCMV-infected cells treated with THAL-SNS032 (0.5µM) or ganciclovir (GCV; 10µM) 2 h p.i., and allowed to migrate for 21 h in transwell inserts 24 h p.i. in the presence of compounds. **(B)** Cell migration was analysed in comparison to mock-infected cells or **(C)** untreated HCMV-infected cells. Experiments were performed in biological duplicates with data representing mean ± SD. ****p <0.0001
